# A Typical Hospital-Acquired Methicillin-Resistant *Staphylococcus aureus* Clone Is Widespread in the Community in the Gaza Strip

**DOI:** 10.1371/journal.pone.0042864

**Published:** 2012-08-16

**Authors:** Asaf Biber, Izeldeen Abuelaish, Galia Rahav, Meir Raz, Liran Cohen, Lea Valinsky, Dianna Taran, Aviva Goral, Abedalla Elhamdany, Gili Regev-Yochay, for the PICR Study Group

**Affiliations:** 1 Infectious Disease Unit, Sheba Medical Center, affiliated to the Sackler School of Medicine, Tel Aviv University, Ramat Gan, Israel; 2 Infectious Disease Epidemiology Section, The Gertner Institute for Epidemiology and Health Policy Research, Ramat Gan, Israel; 3 Global Health Division-Dala Lana School of Public Health, University of Toronto, Toronto, Ontario, Canada; 4 Jerusalem-Hashfela District, Maccabi Healthcare Services, Modiin, Israel; 5 Government Central Laboratories, Ministry of Health, Jerusalem, Israel; 6 Central Laboratory, Maccabi Healthcare Services, Rehovot, Israel; 7 Keystone Consulting Services, Gaza City, the Gaza Strip, Palestine; Rockefeller University, United States of America

## Abstract

Epidemiological data on community acquired methicillin-resistant-*Staphylococcus aureus* (CA-MRSA) carriage and infection in the Middle-East region is scarce with only few reports in the Israeli and Palestinian populations. As part of a Palestinian-Israeli collaborative research, we have conducted a cross-sectional survey of nasal *S. aureus* carriage in healthy children and their parents throughout the Gaza strip. Isolates were characterized for antibiotic susceptibility, *mec* gene presence, PFGE, *spa* type, SCC*mec*-type, presence of PVL genes and multi-locus-sequence-type (MLST). *S. aureus* was carried by 28.4% of the 379 screened children-parents pairs. MRSA was detected in 45% of *S. aureus* isolates, that is, in 12% of the study population. A single ST22-MRSA-IVa, *spa* t223, PVL-gene negative strain was detected in 64% of MRSA isolates. This strain is typically susceptible to all non-β-lactam antibiotics tested. The only predictor for MRSA carriage in children was having an MRSA carrier-parent (OR = 25.5, P = 0.0004). Carriage of the Gaza strain was not associated with prior hospitalization. The Gaza strain was closely related genetically to a local MSSA *spa* t223 strain and less so to EMRSA15, one of the pandemic hospital-acquired-MRSA clones, scarcely reported in the community. The rapid spread in the community may be due to population determinants or due to yet unknown advantageous features of this particular strain.

## Introduction

*Staphylococcus aureus* is a leading cause of human bacterial infection worldwide [1]. Nasal carriage is a major source of endogenous infection as well as of human to human transmission. Methicillin-Resistant *S. aureus* (MRSA) is a major problem in health-care facility settings (HA-MRSA). In the last two decades, community-acquired (CA-MRSA) infections have emerged as well, mainly in young healthy individuals [2]. CA-MRSA strains appear to be more virulent than HA-MRSA strains [3], [4], yet, they are more susceptible to non-β-lactam antibiotic groups [5].

While a few pandemic clones cause most HA-MRSA infections [1], a considerably diverse group of distinct CA-MRSA strains has been documented. Yet, three predominant CA-MRSA clones emerged: MRSA-ST8-IVa(USA300), causing a major epidemic in the USA , MRSA-ST30-IV in Asia and Oceania and MRSA-ST80-IV in Europe, causing mostly sporadic infections with a few local outbreaks [6].

Epidemiological data on CA-MRSA carriage and infection in the Middle-East region is scarce. Only rare cases were found in Israeli [7], [8], or Palestinian populations [9]. In 2009, we established the Palestinian-Israeli collaborative research (PICR) group, in order to explore important infectious disease issues in both Palestinian and Israeli communities.

The Gaza strip is a 360-km^2^ narrow area located on the Eastern coast of the Mediterranean Sea, bordering Egypt on the south, the Mediterranean on the west and Israel on the north and east. It is populated by ∼1.5 million inhabitants. Annual population growth rate is 3.3%, and infant mortality rate is ∼20/1000 (UNRWA data: http://unispal.un.org/UNISPAL.NSF/0/885BD85F 892778F28525772700503A4B).

In this study we report widespread CA-MRSA carriage of a single unique strain among children and their parents throughout different districts of the Gaza strip.

## Methods

### Institutional Review Board (IRB) and Patient Consent

IRB approval was given by the Sheba Medical Center's IRB as well as by a local ethics committee of the Health Ministry in the Gaza strip. Written informed consent was received for each participating individual before recruitment.

### Study period & population

Between March and July 2009, healthy children younger than 5.5 years were randomly selected, using a simple random scheme, in 12 Gaza neighborhoods and villages in northern and central Gaza strip. Children and one of their parents were enrolled following parental signed informed consent. Participants were screened in their homes, nasal swabs were obtained and a questionnaire addressing demographic and medical history information was filled.

### *S. aureus* detection and antibiotic susceptibility testing

Swabs were collected from both anterior nares using a cotton-tipped polyester-swab placed in Amies transport-medium (Copan, Brescia, Italy). All swabs were transmitted to the central Maccabi-Healthcare-Services Laboratory within 24 hours, were plated on tryptic soy agar plates supplemented with 5% sheep blood (HyLabs, Rehovot, Israel) and incubated overnight at 35°C. *S. aureus* was identified by colony morphology, production of catalase, DNAse, and coagulase. Antibiotic susceptibilities were determined by the VITEK-2 system, using plate AST P536 (bioMe'rieux, Hazelwood, MO) for oxacillin, cefoxitine, erythromycin, clindamycin, inducible clindamycin, fusidic acid, gentamicin, trimethoprin-sulfamethoxazole, minocycline, ciprofloxacin and vancomycin. Ciprofloxacin and trimethoprin-sulfamethoxazole susceptibility were also assessed by disc-diffusion and E-test when VITEK-2 results were not available or when susceptibility by VITEK-2 was defined as resistant. Oxacillin resistance was also correlated with *mecA* gene presence by PCR.

### Pulse field gel electrophoresis (PFGE)

Pulse field gel electrophoresis (PFGE) was performed on all MRSA isolates and on 40 randomly selected MSSA isolates. Briefly, *Sma*I digested DNA embedded in agarose plugs were subjected to PFGE analysis at 14°C in a CHEF-DR-III system (Bio-Rad) at, 6 V/cm for 19 h; initial pulse, 2 s; final pulse, 54 s; angle, 120°; in a 0.5×Tris-borate-EDTA buffer. PFGE profiles were analyzed by BioNumerics software v6.5 (Applied Maths, Sint-Martens Latem, Belgium) using the dice coefficient with a 1.5% position tolerance and 1% optimization value. Cluster analysis was performed by the un-weighted pair-group mean analysis (UPGMA). Similarity of 80% of Dice coefficients was defined as PFGE cluster.

### Multilocus sequence typing (MLST)

At least one isolate from each PFGE pattern and at least two from each PFGE cluster were analyzed by MLST. Briefly, isolates were grown on blood agar overnight. Several colonies were re-suspended in 400 µl lysis solution (0.7 µl lysozyme 5 mg/ml, 7 µl lysostaphin 0.5 mg/ml, 4 µl Tris 1 M, 8 µl EDTA 0.5 M, 380 µl H_2_Ox2) and incubated at 37°C for 30 minutes followed by heating at 95°C for 10 minutes. PCR amplification was carried out according to the MLST website (http://www.mlst.net/). PCR products were sequenced and analyzed by the BioNumerics software. Clonal complex (CC) was determined using the program eBURST v3 based on related STs (http://eburst.mlst.net/).

### Spa typing

PCR amplification was performed as described previously [10]. The PCR Products were purified with the Gene JET PCR DNA Purification kit (Fermentas, Burlington city, Ontario, Canada) and sequenced with the Big Dye Terminator v1.1 Cycle Sequencing kit (Applied Biosystems Warrington, UK).The sequencing products were purified with Big Dye XTerminator Purification kit (Applied Biosystems, Warrington, UK) and finally were sequenced using 3100 Avant Genetic Analyzer (ABI, Foster City, CA) and analyzed using the BioNumerics software.

### SCCmec typing

SCCmec typing was performed using the multiplex-PCR assay described by Zhang *et al* [11] with some modifications. Briefly, DNA was extracted by rapid DNA extraction from a single colony suspended in 50 µl of sterile distilled water which was heated at 99°C for 10 min or by the ZR Fungal/Bacterial DNA Mini Prep (Zymo Research, Orange, CA). The cycling parameters were, 94°C for 5 min (or 15 min); 10 cycles of 94°C for 45 s, 60°C for 45 s, 72°C for 90 s; another 25 cycles of 94°C for 45 s, 52°C for 45 s, and 72°C for 90 s, and 72°C for 10 min. As not all SCC*mec* types and subtypes could be determined initially, *ccr* gene complex (types 1,2,3 and 5) and *mec* gene complex (class A, B and C) were assessed [11].

### Panton Valentine Leukocidin

Detection of Panton Valentine Leukocidine (PVL) genes was performed using primers previously described [4]. This was performed for all MRSA and for the 40 MSSA isolates described above.

### Statistical methods

Multivariate logistic regression models were used to assess predictors for *S. aureus* and MRSA carriage among parents and children. Covariates included: age, sex, recent hospitalization, recent antibiotic use, number of household members and being a pet owner (cat, dog, horse or other). Additional covariates assessed as potential predictors for child *S. aureus* carriage were day care attendance and parental *S. aureus* carriage; additional covariates in the parent *S. aureus* carriage model were child *S. aureus* carriage and frequent contact with elderly. MRSA carriage was assessed among *S. aureus* carriers. Covariates significant at p<0.2 in the univariate models were included in the multivariate analysis. To assess genetic diversity, the Simpson's index of diversity (SID) with a confidence interval of 95% was used [12]. The SID was estimated by the combination of the results obtained by PFGE, presence of PV- genes and SCC*mec* type. SAS 9.2 was used.

## Results

### Study population

A total of 379 pairs of children and parents were enrolled (**Table 1**). The median children's age was 1.8 years (range: 3 weeks to 5.5 years); the median parents' age was 32 (range: 19.5–58 years). Among children both genders were equally represented (49.3% vs. 50.7%, male:female). Of the parents, 68.9% were mothers, among which 94% reported to be housewives. Of the fathers, 37% reported to be unemployed. Most of the screened children (93%) had siblings, with a median number of household members of seven (range:2–25). Only 12 (3.4%) children reported attending day care centers. All participants were reported to be healthy and none of them reported a skin infection on the day of screening.

**Table 1:** Predictors for *S. aureus* and MRSA acquisition among children in Gaza, by multivariate analyses.

MRSA carriage		*S. aureus* carriage		n (total)	Variable
aOR$(95% CI);p	n (%**)	aOR* (95% CI);p	n (%)		
	50 (46.7)		107(28.2)	379	All
Age (months)					
Ref	6 (75.0)	Ref	8 (26.7)	30	*<6*
0.40(0.10–1.53);0.18	5 (33.3)	0.89(0.32–2.46);0.81	15 (25.0)	60	*6–11*
0.35(0.11–1.18);0.09	8 (32.0)	0.79(0.31–2.04);0.63	25 (21.7)	115	*12–23*
1.14(0.36–3.54);0.83	14 (53.8)	1.46(0.56–3.82);0.44	26 (34.7)	75	*24–35*
0.96(0.32–2.93);0.95	17 (51.5)	1.36(0.53–3.52);0.52	33 (33.3)	99	*36–66*
					sex
Ref	24 (46.2)	Ref	52 (27.8)	187	*Male*
1.28(0.67–2.44);0.45	26 (47.3)	1.05(0.66–1.68);0.84	55 (28.6)	192	*Female*
Number of household members					
	2 (40.0)	Ref	5 (18.5)	27	*2–3*
	30(51.7)	1.49(0.52–4.31);0.46	58 (27.1)	214	*4–8*
	18(40.9)	1.86(0.63–5.52);0.26	44 (32.1)	137	*9+*
Attend Day care					
		Ref	101 (27.6)	366	*No*
		1.95(0.61–6.27);0.26	6 (46.2)	13	*Yes*
Parental *S. aureus/*MRSA carriage+					
Ref	27 (40.3)	Ref	67 (25.0)	268	*No*
6.30(3.01–13.18);<0.001	22(56.4)	1.73(1.05–2.86);0.03	39 (36.1)	108	*Yes*
Cat owner					
		Ref	86 (26.3)	327	*No*
		2.11(1.11–4.00);0.02	21 (40.4)	52	*Yes*

* Multivariate model for child *S. aureus* carriage included: being a cat owner, parental *S. aureus* carriage, day care center attendance, number of household members, child sex and age.

** % from *S. aureus carriers*.

$ Multivariate model for child MRSA carriage included: child age, sex and parental MRSA carriage.

+ Models adjusted as described above.

### *S. aureus* and MRSA carriage among children (Table 1)

Of the children, 107/379 (28.2%) were *S. aureus* carriers. MRSA was detected in 50 children (46.7% of *S. aureus* carriers and 13.2% of all children). Predictors for *S. aureus* carriage in children were having a *S. aureus* carrier parent (aOR = 1.73, 95% CI 1.05–2.86;P = 0.03) and being a cat owner (aOR = 2.11, 95% CI 1.11–4.00;P = 0.02). The only predictor for MRSA carriage in children was having a MRSA carrier parent (aOR = 6.3, 95% CI 3.01–13.18;P = <0.001).

### *S. aureus* and MRSA carriage among parents (Table 2)

**Table 2:** Predictors for *S. aureus* and MRSA acquisition among parents in Gaza, by multivariate analyses.

MRSA carriage		*S. aureus* carriage		n (total)	Variable
aOR$ (95% CI);p	n, (%**)	aOR*(95% CI);p	n (%)		
	44 (40.7)		108 (28.5)	379	All
1.00(0.95–1.04);0.87+		0.96(0.93–0.99);0.02+			Age (years)
Ref	7 (31.8)	Ref	22 (35.5)	62	*<25*
0.88(0.33–2.35);0.80	20 (37.0)	0.85(0.44–1.65);0.63	54 (32.9)	164	*25–34*
1.05(0.38–2.91);0.92	15 (53.6)	0.48(0.23–0.99);0.05	28 (21.9)	128	*35–44*
0.92(0.15–5.74);0.93	2 (66.7)	0.45(0.11–1.94);0.29	3 (23.1)	13	*45+*
Sex					
Ref	10 (43.5)	Ref	23 (21.3)	108	*Male*
1.33(0.60–2.94);0.48	33 (40.2)	1.48(0.84–2.62);0.18	82 (34.2)	240	*Female*
Hospitalization in the previous 6 months					
		Ref	107 (30.1)	356	*No*
		0.08(0.01–0.64);0.02	1 (4.30)	23	*Yes*
					*Pet owner*
		Ref	76 (26.2)	290	*No*
		1.27(0.73–2.21);0.39	32 (36.4)	88	*Yes*
*Child S. aureus/MRSA carriage* ++					
	2 (11.8)	Ref	69 (25.4)	272/17	*No*
6.38(3.05–13.32);<0.0001	17 (77.3)	1.78(1.05–3.04);0.03	39 (36.4)	107/22	*Yes*

* Multivariate model for parent *S. aureus* carriage included: age, sex, recent hospitalization, pet owner and child *S. aureus* carriage.

** % from *S. aureus carriers*.

$ Multivariate model for parent MRSA carriage included: age, sex and child MRSA carriage.

+ aOR for continuous parental age.

++ Models adjusted as described above.

Of the parents 108/379 (28.5%) were *S. aureus* carriers, and 44 (40.7% of carriers and 11.6% of all parents) were MRSA carriers. The variables significantly associated with *S. aureus* carriage among parents were younger age (aOR 0.96, 95%CI 0.93–0.99 per year; p = 0.02), no previous hospitalization (aOR 0.08, 95%CI 0.01–0.64;p = 0.02) and *S. aureus* carriage by the child (aOR 1.78, 95%CI 1.05–3.04;p = 0.03). The only predictor for MRSA carriage among parents was having a MRSA carrier child (aOR 6.38, 95%CI 3.05–13.32; p<0.001).

### Characteristics of the MRSA clones

The 94 MRSA isolates identified were further characterized by PFGE, SCC*mec* typing, *spa* typing and presence of PVL genes. Twenty-seven isolates were subject to MLST typing, 15 of these were ST22. Characterization of the isolates is presented in **Table 3**. Overall, a low genetic diversity of MRSA clones, as determined by combining the results obtained by PFGE, SCC*mec* typing and presence of genes encoding PVL was observed (SID 0.58, 95%CI 047–0.70). The predominant clonal complex, CC22 was found in 70 strains, 74.5% of all MRSA isolates. The genetic diversity of MRSA belonging to this clonal complex was relatively low (SID 0.28, 95%CI 0.15–0.41). Moreover, a single strain, as determined by the Simpson's index (ST22-MRSA-IVa, negative for PVL -genes and of a single PFGE cluster) was detected in 60/94 (64%) cases and defined here as the Gaza strain. Other clonal complexes detected were mainly CC88 (n = 7, 7.4%) and CC80 (n = 5, 5.3%).

**Table 3:** Molecular characteristics of 94 MRSA isolates.

CC*	MLST*	SCC*mec*	PVL-genes	n	Antibiotic susceptibility profile						
					cip	min	tri	Gen	fus	cli	ery
CC22 (n = 71)	ST22 (69)	IVa (62)	neg (60)	37	S	S	S	S	S	S	S
				23	S	S	S	S	S	R	R
			pos (2)	2	S	S	S	S	S	S	S
		V (7)	neg	4	S	S	S	S	S	S	S
				2	S	S	S	R	S	S	S
				1	S	S	S	I	S	S	S
	ST1784 (2)	V	neg	2	S	S	S	S	S	S	S
CC88 (n = 7)	ST78	IVa	neg	5	S	S	S	S	S	R	R
				1	R	S	S	S	S	R	R
				1	S	S	S	S	S	S	S
CC80 (n = 5)	ST80	IVa	pos	4	S	S	S	S	I	S	S
		IV not a–d	pos	1	S	S	S	S	I	S	S
CC5 (n = 3)	ST5 (1)	V	neg	1	S	S	S	S	S	R	R
	ST1785 (2)	IVa	neg	2	S	R	S	S	S	R	R
CC30 (n = 2)	ST30	IVc	pos	1	S	S	S	S	S	R	R
	ST1734	IVc	neg	1	S	S	S	S	S	S	S
CC913 (n = 1)	ST913	IVa	neg	1	S	S	S	S	S	R	R
Others (n = 5)			neg	5							

* MLST and CC was deducted from PFGE pattern after at least one isolate representative of each PFGE pattern was submitted to MLST typing.

n-numer of isolates,ery- erythromycin, cli- clindamycin, fus- fusidic acid, gen- gentamicin, tri- trimethoprin/sulfamethoxazole, min- minocycline, R-resistance, I- intermediate resistance, S- susceptibility.

While the SCC*mec* type of the Gaza strain was IVa, nine closely related isolates (by PFGE) carried SCC*mec* type V; seven of these also belonged to ST22 and two isolates belonged to a novel single locus variant of ST22: ST1784.

Of all 94 MRSA isolates, eight (8.5%) were positive for PVL-genes; Five PVL-gene positive isolates belonged to a single ST80-MRSA-IVstrain, a single ST30MRSA-IVc PVL-gene positive isolate was detected, and two isolates of the Gaza strain PFGE cluster were PVL-gene positive. The *spa* type of the Gaza strain as well as that of the closely related strains (the ST22-MRSA-IVa- PVL-gene positive, as well as the ST22-MRSA-V strains) were all *spa* type t223.

**Figure 1:**
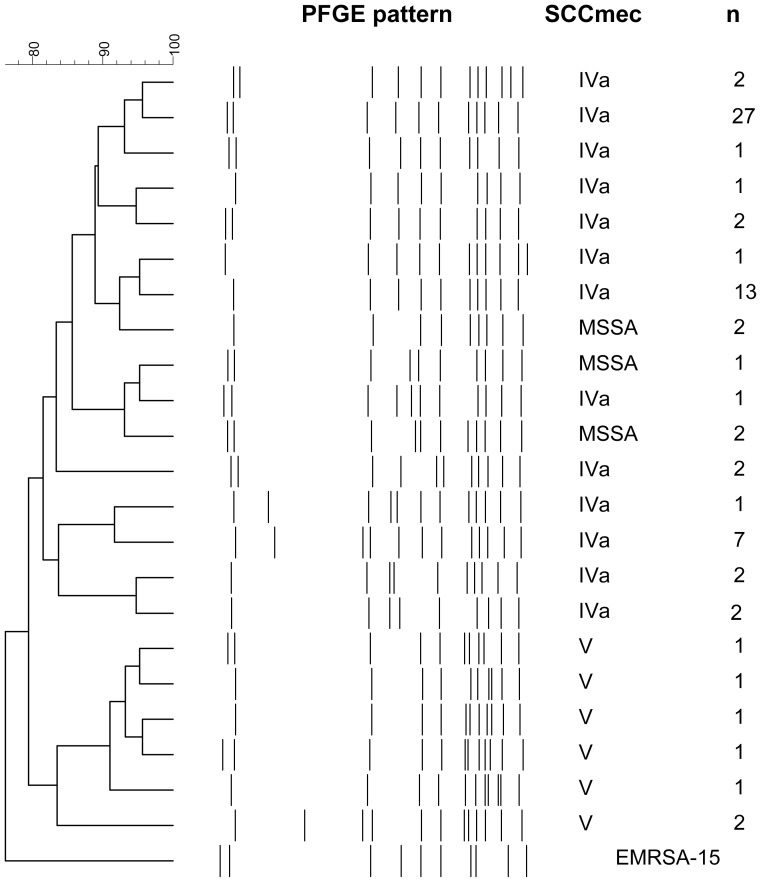
Molecular relatedness of ST22 isolates in this survey. Dendrogram of all isolates corresponding to ST22, by deduction from PFGE patterns (see Methods), including MRSA SSC*mec* IV and V, MSSA and EMRSA-15 isolate as a reference strain.

The Gaza strain consisted of 13 closely related PFGE patterns with two predominant patterns, all with similarity of more than 81% as determined by the Dice coefficients. This strain is genetically related to the epidemic HA-MRSA-ST22 clone, EMRSA-15, with 76% PFGE pattern similarity (**Figure 1**).

#### Antibiotic susceptibility

All MRSA isolates were vancomycin, trimethoprim/sulfametoxazole and ciprofloxacin susceptible excluding a single isolate of ST78-MRSA which was ciprofloxacin resistant and had inducible resistance to clindamycin. Interestingly, 27% of the Gaza strain isolates were initially reported as trimethoprim/sulfametoxazole resistant by VITEK-2. Due to previous reports of inaccuracy in detecting susceptibility by the automatic systems [13], we also tested these strains using the disk diffusion and E-test methods and finally defined them as susceptible. Erythromycin resistance was observed in 37.9% of cases and all had inducible clindamycin resistance. Aminoglycoside resistance was detected in 3/94 isolates. Most CC22 isolates carrying SCC*mec* IV were susceptible to all non-β-lactam classes. Similarly, most CC22 isolates carrying SCC*mec* type V were susceptible to all non-β-lactam classes, however 3/9 (33%) were non-susceptible to gentamicin.

#### Predictors for carriage

We could not identify any predictors for carriage of ST22-MRSA-IVa; it was isolated from all villages and neighborhoods that were sampled in this survey and from both children (n = 34, 55%) and parents (28, 45%). Only 7 (11.3%) of the carriers of this strain had a history of previous hospitalization (similar to MSSA and non-*S. aureus* carriers).

**Figure 2:**
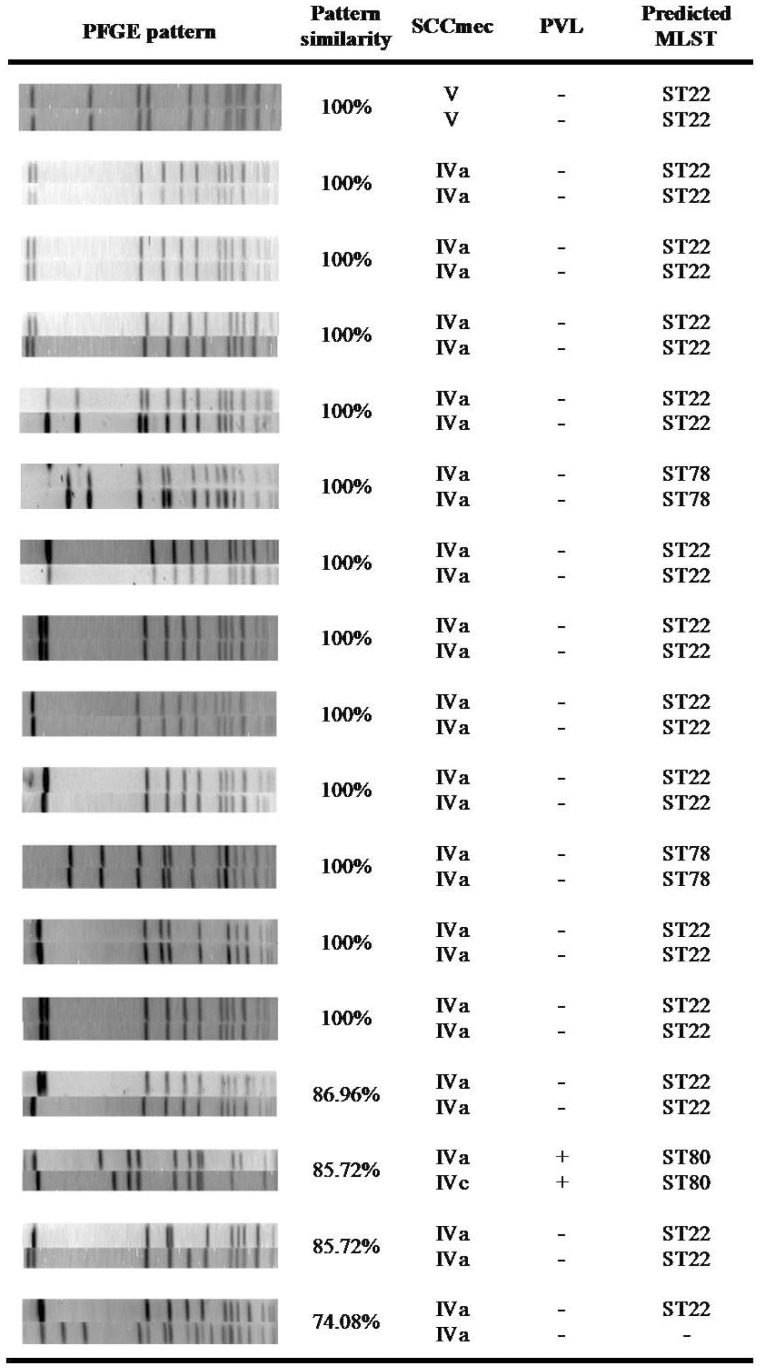
Molecular characterization and relatedness of MRSA isolates carried by parent-child pairs.

Since the only predictor for MRSA carriage by a child was having a MRSA-carrier parent (88.9% versus 39.6% carriage rates in children of MRSA-carrier parents vs. non-MRSA carriers (p<0.0001), we assessed the genetic relatedness of MRSA isolates from parents and their children. In seventeen child-parent couples (36.1% of all MRSA carriers), both the child and his/her parent were MRSA carriers (**Figure 2**). 13/17 (82.35%) carried an identical strain.

### Characteristics of the MSSA clones

**Figure 3:**
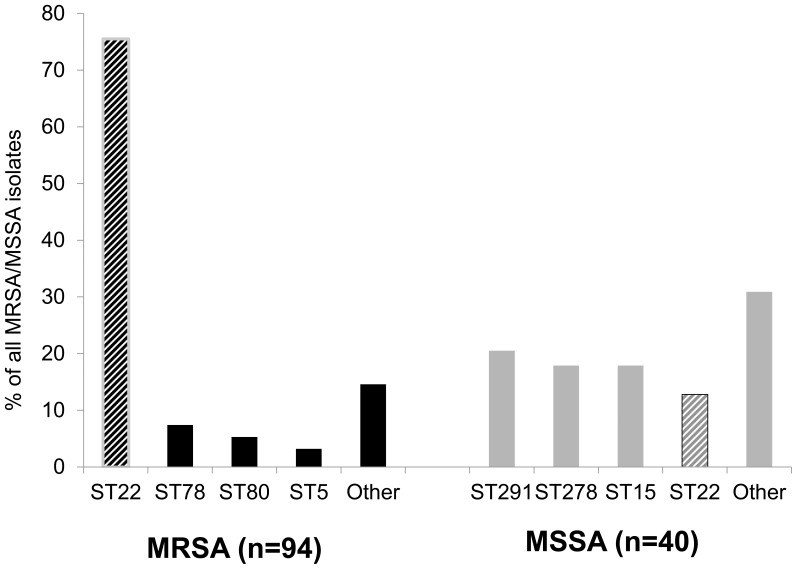
Distribution of MLSTs among MRSA and MSSA isolates tested. Black – MRSA, Grey- MSSA, striped – ST22.

Since the origin of the predominant MRSA clone could have originated from a widespread MSSA clone, we assessed the genetic relatedness of 40 randomly chosen MSSA (**Figure 3**) isolates. Three main clones were identified; ST291in 8 (20.5%) individuals, ST1278 in 7 (17.9%) individuals and ST15 in 7 (17.9%) individuals. A single ST22 strain was detected in five individuals (12.8%), 3 children and 2 unrelated parents. This strain was closely related to the ST22-MRSA-IVa clone (over 80% similarity by PFGE), also of *spa* t223 (**Figure 1** **)**. None of the ST22-MSSA isolates carried the PVL genes. A single ST121-MSSA isolate was positive for genes encoding PVL.

## Discussion

We report widespread CA-MRSA carriage of a single strain in the Gaza strip. The Gaza strain belongs to CC22 and is genetically related to the epidemic hospital EMRSA15 clone.

In this study, nearly 30% of healthy children and parents carried *S*. *aureus*, similar to reports from other geographical regions [14]. Yet, MRSA carriage rate was dramatically higher (12.4% of the study population). This prevalence rate is much greater than any report from the region [7], [8], including a report on CA-MRSA in the West Bank [9]. Predictors for *S. aureus* carriage were living in large families, owning pet cats and *S. aureus* carriage by another family member. Yet, the only predictor for MRSA carriage was MRSA carriage by another family member.

The most common ST22-MRSA clone worldwide is the epidemic HA-MRSA EMRSA-15, which emerged in the UK in 1991. Since then, it has become one of the dominant strains in that region and is currently considered one of the global HA-MRSA pandemic clones [15]. While EMRSA-15 is regarded as a typical HA-MRSA strain, it was recently reported to be carried asymptomatically by a few (5/879) healthy individuals without associated health-care risk factors [16] [23]. Another recent study reported EMRSA-15 in urban Portuguese public buses [17]. Yet, a few key features differentiate EMRSA-15 from the Gaza strain; While EMRSA-15 is known to be ciprofloxacin resistant [15] the Gaza strain is ciprofloxacin susceptible, EMRSA-15 contains SCC*mec*-IVh (vs. SCC*mec*-IVa) and its common *spa* types are t032 and t022 [18] (vs. t223). The quinolone-susceptibility difference may be due to infrequent quinolone use in the Gaza strip [IA personal communication] and does not necessarily imply evolutionary difference between the Gaza strain and EMRSA-15.

Another hospital acquired ST22 clone, referred to as the Barnim epidemic-MRSA strain, considered ancestral to EMRSA-15 in Germany, contains SCC*mec*-IVa. This clone emerged in three hospitals in north Berlin in 1996 and spread to other healthcare facilities throughout Germany. Like EMRSA-15, the Barnim strain is ciprofloxacin resistant and has not yet been reported in the community [19], [20].

Interestingly, EMRSA-15 has been reported to be a common clone isolated from small companion animals, specifically dogs and cats, in the community [21]. We found that owning a cat was associated with increased *S. aureus* carriage, but not particularly MRSA carriage.

The origin of the Gaza clone could either be an HA-MRSA that spread into the community, or alternatively a local ST22-MSSA that evolved into a novel CA-MRSA clone. While previous hospitalization was relatively frequent (13.7%) it was not a predictor for carrying this strain or MRSA in general. Furthermore, our data is suggestive of local evolution rather than import of EMRSA-15 as implied by the high genetic relatedness of the Gaza strain to the ST22-MSSA-*spa*-t223 strains in the region. Further thorough genomic studies of these strains would be required to conclusively determine its evolution.

The ST22-MRSA-V clone, which is closely related to the predominant strain; with ∼80% similarity by PFGE and of the same *spa* type, was detected in only 7/94 (7.44%) vs. 61/94 (64.89%) of ST22-MRSA-IVa isolates. ST22-MRSA-V may have evolved from the same ancestor or alternatively could have evolved from the Gaza-strain, by acquiring SCC*mec*-V cassette after losing the IVa cassette, as has been suggested for ST8-MRSA strains [22].

The striking fact that a single strain is attributed to over 60% of all MRSA isolates may suggest that specific yet unknown features of this strain confer advantage over competing MRSA and MSSA strains. The relatively lower proportion of ST22 among MSSA isolates as compared to that among MRSA is supportive of this idea. However, we cannot rule out the possibility that the overcrowding in this region facilitated spread of a coincidental, typically nosocomial strain in the community.

Furthermore, intrafamilial MRSA transmission has been implicated to play an important role in MRSA transmission [23]. Given the large number of household members and the fact that only two family-members were sampled, the intrafamilial MRSA transmission reported in this study was probably under-estimated. It is well known that crowded living conditions contribute to CA-MRSA transmission, as reported among athletes, prisoners and military trainees [24], [25], [26] as well as among minorities and developing populations [27].

Yet, the fact that a single MRSA strain became so predominant, while total *S. aureus* carriage rate was not different from that in other regions, suggests the Gaza strain has advantages over other local MRSA strains. Further studies to determine the unique characteristics of this strain are needed.

## References

[1] Chambers HF, Deleo FR, editors (2009) Waves of resistance: Staphylococcus aureus in the antibiotic era. 629–641. p. 10.1038/nrmicro2200 PMC2871281 19680247

[2] Enright MC, Robinson DA, Randle G, Feil EJ, Grundmann H, et al (2002) The evolutionary history of methicillin-resistant Staphylococcus aureus (MRSA). Proc Natl Acad Sci U S A 99: 7687–7692. 12032344 10.1073/pnas.122108599 PMC124322

[3] Gillet Y, Issartel B, Vanhems P, Fournet JC, Lina G, et al (2002) Association between Staphylococcus aureus strains carrying gene for Panton-Valentine leukocidin and highly lethal necrotising pneumonia in young immunocompetent patients. Lancet 359: 753–759. 11888586 10.1016/S0140-6736(02)07877-7

[4] Lina G, Piemont Y, Godail-Gamot F, Bes M, Peter MO, et al (1999) Involvement of Panton-Valentine leukocidin-producing Staphylococcus aureus in primary skin infections and pneumonia. Clin Infect Dis 29: 1128–1132. 10524952 10.1086/313461

[5] Diep BA, Chambers HF, Graber CJ, Szumowski JD, Miller LG, et al (2008) Emergence of multidrug-resistant, community-associated, methicillin-resistant Staphylococcus aureus clone USA300 in men who have sex with men. Ann Intern Med 148: 249–257. 18283202 10.7326/0003-4819-148-4-200802190-00204

[6] Tristan A, Bes M, Meugnier H, Lina G, Bozdogan B, et al (2007) Global distribution of Panton-Valentine leukocidin–positive methicillin-resistant Staphylococcus aureus, 2006. Emerg Infect Dis 13: 594–600. 17553275 10.3201/eid1304.061316 PMC2725977

[7] Adler A, Givon-Lavi N, Moses AE, Block C, Dagan R (2009) Carriage of community-associated methicillin-resistant Staphylococcus aureus in a cohort of infants in southern Israel: risk factors and molecular features. J Clin Microbiol 48: 531–538. 20007386 10.1128/JCM.02290-08 PMC2815591

[8] Regev-Yochay G, Carmeli Y, Raz M, Pinco E, Etienne J, et al (2006) Prevalence and genetic relatedness of community-acquired methicillin-resistant Staphylococcus aureus in Israel. Eur J Clin Microbiol Infect Dis 25: 719–722. 17043835 10.1007/s10096-006-0210-3

[9] Kaibni MH, Farraj MA, Adwan K, Essawi TA (2009) Community-acquired meticillin-resistant Staphylococcus aureus in Palestine. J Med Microbiol 58: 644–647. 19369527 10.1099/jmm.0.007617-0

[10] Shopsin B, Gomez M, Montgomery SO, Smith DH, Waddington M, et al (1999) Evaluation of protein A gene polymorphic region DNA sequencing for typing of Staphylococcus aureus strains. J Clin Microbiol 37: 3556–3563. 10523551 10.1128/jcm.37.11.3556-3563.1999 PMC85690

[11] Zhang K, McClure JA, Elsayed S, Louie T, Conly JM (2005) Novel multiplex PCR assay for characterization and concomitant subtyping of staphylococcal cassette chromosome mec types I to V in methicillin-resistant Staphylococcus aureus. J Clin Microbiol 43: 5026–5033. 16207957 10.1128/JCM.43.10.5026-5033.2005 PMC1248471

[12] Simpson EH (1949) Measurement of species diversity. Nature 163: 688.

[13] Carmichael IC, Godfrey V, Nicholson G (1999) Errors associated with determining the susceptibilities of staphylococci to trimethoprim by the Vitek GPS-AK card. J Antimicrob Chemother 44: 293–294. 10473241 10.1093/jac/44.2.293

[14] Wertheim HF, Melles DC, Vos MC, van Leeuwen W, van Belkum A, et al (2005) The role of nasal carriage in Staphylococcus aureus infections. Lancet Infect Dis 5: 751–762. 16310147 10.1016/S1473-3099(05)70295-4

[15] O'Neill GL, Murchan S, Gil-Setas A, Aucken HM (2001) Identification and characterization of phage variants of a strain of epidemic methicillin-resistant Staphylococcus aureus (EMRSA-15). J Clin Microbiol 39: 1540–1548. 11283084 10.1128/JCM.39.4.1540-1548.2001 PMC87967

[16] Mollaghan AM, Lucey B, Coffey A, Cotter L (2010) Emergence of MRSA clone ST22 in healthy young adults in the community in the absence of risk factors. Epidemiol Infect 138: 673–676. 20144250 10.1017/S0950268810000191

[17] Simoes RR, Aires-de-Sousa M, Conceicao T, Antunes F, da Costa PM, et al (2011) High prevalence of EMRSA-15 in Portuguese public buses: a worrisome finding. PLoS One 6: e17630. 21407807 10.1371/journal.pone.0017630 PMC3047573

[18] Milheirico C, Oliveira DC, de Lencastre H (2007) Multiplex PCR strategy for subtyping the staphylococcal cassette chromosome mec type IV in methicillin-resistant Staphylococcus aureus: ‘SCCmec IV multiplex’. J Antimicrob Chemother 60: 42–48 Epub 2007 Apr 2028. 17468509 10.1093/jac/dkm112

[19] Ghebremedhin B, Konig W, Witte W, Hardy KJ, Hawkey PM, et al (2007) Subtyping of ST22-MRSA-IV (Barnim epidemic MRSA strain) at a university clinic in Germany from 2002 to 2005. J Med Microbiol 56: 365–375. 17314368 10.1099/jmm.0.46883-0

[20] Witte W, Enright M, Schmitz FJ, Cuny C, Braulke C, et al (2001) Characteristics of a new epidemic MRSA in Germany ancestral to United Kingdom EMRSA 15. Int J Med Microbiol 290: 677–682. 11310446 10.1016/S1438-4221(01)80006-0

[21] Cuny C, Friedrich A, Kozytska S, Layer F, Nubel U, et al (2009) Emergence of methicillin-resistant Staphylococcus aureus (MRSA) in different animal species. Int J Med Microbiol 300: 109–117. 20005777 10.1016/j.ijmm.2009.11.002

[22] Fontanilla JM, Kirkland KB, Talbot EA, Powell KE, Schwartzman JD, et al (2009) Outbreak of Skin Infections in College Football Team Members Due to an Unusual Strain of Community-Acquired Methicillin-Susceptible Staphylococcus aureus. J Clin Microbiol 48: 609–611. 20007392 10.1128/JCM.02297-09 PMC2815631

[23] Huijsdens XW, van Santen-Verheuvel MG, Spalburg E, Heck M, Pluister GN, et al (2006) Multiple Cases of Familial Transmission of Community-Acquired Methicillin-Resistant Staphylococcus aureus. J Clin Microbiol 44: 2994–2996. 16891525 10.1128/JCM.00846-06 PMC1594612

[24] Campbell KM, Vaughn AF, Russell KL, Smith B, Jimenez DL, et al (2004) Risk factors for community-associated methicillin-resistant Staphylococcus aureus infections in an outbreak of disease among military trainees in San Diego, California, in 2002. J Clin Microbiol 42: 4050–4053. 15364988 10.1128/JCM.42.9.4050-4053.2004 PMC516279

[25] Kazakova SV, Hageman JC, Matava M, Srinivasan A, Phelan L, et al (2005) A clone of methicillin-resistant Staphylococcus aureus among professional football players. N Engl J Med 352: 468–475. 15689585 10.1056/NEJMoa042859

[26] Kajita E, Okano JT, Bodine EN, Layne SP, Blower S (2007) Modelling an outbreak of an emerging pathogen. Nat Rev Microbiol 5: 700–709. 17703226 10.1038/nrmicro1660

[27] Charlebois ED, Bangsberg DR, Moss NJ, Moore MR, Moss AR, et al (2002) Population-based community prevalence of methicillin-resistant Staphylococcus aureus in the urban poor of San Francisco. Clin Infect Dis 34: 425–433 Epub 2002 Jan 2002. 11797167 10.1086/338069

